# Biomaterials in orthopaedics: the past and future with immune modulation

**DOI:** 10.1186/s40824-020-0185-7

**Published:** 2020-02-04

**Authors:** Gun-Il Im

**Affiliations:** Research Institute for Integrative Regenerative Biomedical Engineering, Dongguk University Ilsan Hospital, 814 Siksa-Dong, Goyang, 410-773 Republic of Korea

**Keywords:** Orthopaedics, Biomaterials, History, Immune response, Perspective

## Abstract

Orthopaedics owes its current status of advanced care to the development of biomaterial science more than any other clinical medical specialty. The purpose of this brief review is to introduce the history and present status of biomaterials in orthopaedic field and cast a perspective on the future use of biomaterials to treat musculoskeletal disorders with particular emphasis on immune modulation. While the biomaterials in orthopaedics started from inert materials to replace the function and structure of hard tissue such as bone and cartilage, regenerative medicine will play a greater role in preventing the traumatic loss of tissues, as well as in the earlier stages of diseases. The understanding and modulation of immune response to biomaterials will further lead to the better incorporation of implants into host tissue or the near-perfect regeneration of host tissue.

## Introduction

A biomaterial is a substance that has been engineered to take a form which, alone or as part of a complex system, is used to direct, by control of interactions with components of living systems, the course of any therapeutic or diagnostic procedure [1]. Orthopaedics, which is a branch of clinical medicine that specializes in the diagnosis and treatment of musculoskeletal disease and trauma in the spine and extremities, owes its current status of advanced care to the development of biomaterial science more than any other clinical medical specialty. Biomaterials can be used to restore or augment the physiological function of diseased or damaged tissues via tissue replacement or regeneration in orthopaedics [2]. The purpose of this brief review is to introduce the history and present status of biomaterials in orthopedic field and cast a perspective on the future use of biomaterials to treat musculoskeletal disorders.

## History and current application of biomaterials in orthopaedics

Orthopaedics started in the 18th century as a group of techniques which used non-surgical means to correct deformities in growing children. At that time, surgical treatment of bone and joint disorders was not possible due to the lack of antiseptic methods and anesthesia which would make operation safe and endurable. Development of inhalation anesthesia and antiseptic methods in 19th century made general surgery available for suffering patients. On the other hand, a lack of suitable biocompatible material prevented bone and joint surgery from being an option in deformity correction or fracture management until the early 20th century.

The development of metallic engineering in last century produced various biocompatible alloys, including stainless steel. Plates, screws, and nails that can be used to fix the bone were devised using those materials, which revolutionized fracture care. Injuries that were once treated by suspending the limb in traction for a month or by wrapping the injury in a heavy cast were now treated by internal fixation. More refined biocompatible metal alloys, such as cobalt chrome alloys, are now the primary material used for artificial joints, which require permanent implantation.

The development of chemical engineering has also produced polymeric material, such as ultra-high molecular weight polyethylene (UHMWPE) or polymethylmethacrylate (PMMA), which have been gradually employed as bearing materials and bonding materials for artificial joints. Bioinert ceramics such as aluminum oxide or zirconium oxide rank as one of hardest materials found in the earth. These materials are now also used as bearing material in joint replacements. While first generation ceramics were fraught with frequent component breakage, the second generation bioinert ceramics, i.e. alumina augmented with zirconia, are now widely used in Korea, and almost replace UHMWPE as the bearing material of choice in total hip arthroplasty. These materials are expected to be used continuously for joint replacement. On the other hand, bioactive ceramics, such as calcium phosphates or calcium sulfates, are used as bone substitutes which fill up bone defects and function as osteo-conductive materials.

Biocompatible & bioabsorbable polymeric materials such as polyglycolic acid (PGA), polylactic acid (PLA), and polydioxanone (PDO) have been used as suture materials for a decade. These materials are now developed into screws, pins, and plates. Furthermore, these absorbable polymers as used as scaffolds for tissue engineering of cartilage and bone.

## Regenerative medicine and immune response in biomaterials

While replacements using biomaterials will continue to be improved upon and will continue to be used to treat advanced diseases at a reduced cost of implanted materials, regenerative medicine will play a greater role in preventing the traumatic loss of tissues, as well as in the earlier stages of diseases. Stem cells, in combination with biomaterials, will be essential for those tissue engineering approaches.

Stem cell–based regenerative approaches have focused on implanting cells that have been seeded or encapsulated in biomaterials. Exogenous stem cell application has not yet proven to be generally effective for the regeneration of most tissues lost by degenerative processes or trauma. While it is expected that these exogenous cells are engrafted into host tissue, most of the cells perish after short period time. Furthermore, inflammation takes place at the site of implantation. As a result, immune response has recently developed into a big issue in the area of tissue engineering [2].

Regenerative power and the development of immune system are inversely related relationship in mammals. Evolutionary and developmental advances in the immune system came with the loss of capacity to fully regenerate damaged tissues [3–5]. Most mammalian tissues do not regenerate themselves. This is related to the their highly developed immune system [6]. In case of tissue damage, resident precursor cell is activated to proliferate, or a scar is formed. Cellular debris is also rapidly cleared to remove potentially toxic or immunogenic materials. Phagocytes are activated to secrete immune-modulatory factors. Macrophages in mammalian cells are in charge of those functions and play a primary role in innate immunity. Interestingly, macrophages show polarized, biphasic responses to tissue injury. Under inflammatory environments, macrophages polarize into classically activated (M1) or alternatively activated (M2) subtypes which differ in their function and marker/cytokine profiles [7]. M1 cells typically produce pro-inflammatory cytokines and nitric oxides for host defense, which can lead to host tissue damage. On the other hand, M2 macrophages secrete anti-inflammatory and immune-modulatory substances, which mediates the resolution of inflammation and the wound healing, causing tissue repair. Timely activation and balance of each macrophage subtype is important for tissue healing. As early infiltration by M1 macrophages clears necrotic tissue [8, 9], disruption of macrophage polarization may impair tissue regeneration [10].

## Immuno-modulating biomaterials

In the future, the concept of modulating an immune response towards the optimal clinical result will be widely applied in orthopaedic biomaterials. Immuno-modulating biomaterials can be broadly categorized into two: (1) biomaterials for replacement that integrate within the body and remain permanently inside on implantation, causing minimal inflammation and fibrous tissue formation; (2) biomaterials for regeneration that offer initial support and stimulate the formation of new tissue but eventually are degraded in a controlled way over time [2].

### Biomaterials for replacement

Biomaterials for replacement are typically long-term (> 20 years) or permanently implantable devices. They are composed of polymers, ceramics, or metals that are very stable mechanically and show minimal host response when implanted [11]. Biologically inert implants that minimize the cell-implant interactions in the microenvironment had been previously preferred [12]. Those implants usually have native proteins adsorbed on the surface, which promote the formation of provisional matrix and function as a buffer between the biomaterial and the host. Also, precise surgical techniques minimize the relative motion between the implant and host tissue [2].

Contrarily, some cell-implant interactions can enhance immune tolerance and integration of implant into host tissue in certain conditions. Titanium implants for joint replacements demonstrate higher osseointegration when the surface is reformed to induce the migration and attachment of osteoblasts [13, 14]. Such alterations may also subsequently induce a pro-M2 polarization, thereby providing a favorable immune environment for bone remodeling. Modifying surface chemistries and roughness can incline the polarization to M2 type, which will in turn lead to greater secretion of regenerative/anti-inflammatory factors and minimize the formation of fibrous tissue [13, 15, 16]. Recent advancement in bioengineering blurs the boundary between replacement and regenerative biomaterials. Numerous coating technologies on replacement implants are functionally analogous to those used for regenerative medicine [2].

### Biomaterials for regeneration

Biomaterials for regeneration aim to restore the lost structure and function of damaged tissue [2]. These materials should degrade in a period spanning several days to months while promoting the regeneration of host tissue that includes the regenerated matrices [15, 17]. The initial M1 response recruits inflammatory cells to the implantation site and instigates the foreign body response [15, 18, 19], which is a necessary early event for wound healing. However, prolonged presence of M1 macrophages leads to the production of cytotoxic reactive oxygen products [20, 21]. Also, fibrous capsule formation from extended inflammation can impair the biomaterials’ capacity to promote tissue formation or hinder its ability to degrade as intended. Thus, a succeeding transition to the M2 type is necessary to promotes tissue remodeling [2].

The anti-inflammatory cytokines (IL-4, IL-10) or small molecules (steroids), can be used to modulate the immune response so that native signaling is overwhelmed and directed to M2 polarization to macrophage [22, 23]. This could be done by incorporating them into controlled release systems [23, 24].

Several biochemical and biophysical properties may be utilized to influence macrophage polarization [25, 26]. The surface topography, including pore size of the scaffold, can be modulated to induce optimal macrophage polarization [27–29]. However, these modification of biomaterial designs to modulate macrophage behavior directly should be done with considerations for undesired effects on other types of cells that contribute to tissue regeneration [2, 30].

## Orthopedic biomaterials in the future

Orthopaedics has benefited from the advances in biomaterials. Orthopaedic biomaterials started from temporary implants to fix bone to permanent implant materials and bioabsorbable implants. In the future, orthopaedic biomaterial will find a place in the regeneration of living tissue, as well as replacing it.

Ongoing research will reveal more details of the inherent qualities of biomaterials and their role in immune-modulation. This insight into biomaterial-immune response interaction will finally lead to an ultimate set of principles and help to create a new group of immuno-modulating biomaterials that can actively direct the innate immune system towards better incorporation of implants into host tissue or the near-perfect regeneration of host tissue.

## Conclusion

While the biomaterials in orthopaedics started from inert materials to replace the function and structure of hard tissue such as bone and cartilage, regenerative medicine will play a greater role in preventing the traumatic loss of tissues, as well as in the earlier stages of diseases. The understanding and modulation of immune response to biomaterials will further lead to the better incorporation of implants into host tissue or the near-perfect regeneration of host tissue.

## Data Availability

Not applicable.

## Abbreviations

pdo: Polydioxanone
pga: Polyglycolic acid
pla: Polylactic acid
pmma: Polymethylmethacrylate
uhmwpe: Ultra-high molecular weight polyethylene
